# The maturation of regional sweating patterns from childhood to young adulthood in females

**DOI:** 10.1113/EP093788

**Published:** 2026-04-11

**Authors:** Hannah Blount, Nuno Koch Esteves, Jade Ward, Grant H Simmons, Peter R Worsley, Davide Filingeri

**Affiliations:** ^1^ ThermosenseLab, Skin Sensing Research Group, School of Health Sciences The University of Southampton Southampton UK; ^2^ PressureLab, Skin Sensing Research Group, School of Health Sciences The University of Southampton Southampton UK; ^3^ Nike Sport Research Lab Nike Inc. Beaverton Oregon USA; ^4^ Tyndall Centre for Climate Change Research The University of Southampton Southampton UK

**Keywords:** female, puberty, skin temperature, sweat, thermal sensation

## Abstract

Children are considered heat vulnerable, with the belief that maturation of sweating occurs throughout childhood. Indeed, young children demonstrate distinct patterns of regional sweating compared to adults, but little is known about this pattern maturation throughout puberty. This study aimed to investigate the maturation of regional sweating patterns and thermal perceptions in females during exercise. Twenty‐eight females aged 8–25 years, representing five Tanner stages (preadolescent to adult) were recruited. Local sweat rates (LSR), sweat output per gland and thermal perceptions were measured during cycling exercise at fixed evaporative requirements for heat balance (154 ± 10 W/m^2^) in a climatic chamber (36°C and 50% relative humidity). Results indicated that LSR across the torso (chest, abdomen, back), but not the limbs (hand, thigh, shin), increased linearly with age, due to increases in sweat output per gland. The transition of regional sweating patterns from children‐like (higher LSR at extremities) to adult‐like (higher LSR at torso) became meaningful (2‐fold difference) at Tanner stage 3 and age 14. Perceptions of temperature, wetness and thermal comfort did not differ across age‐groups. Our findings provide the first detailed evaluation of regional sweating pattern maturation in females while exercising in the heat. This could inform person‐centred public health and sportswear applications.

## Abstract (highlights)

- **What is the central question of this study?**
  How and when do regional sweating patterns mature in females during puberty, and do these physiological changes affect thermal perceptions in heat?
- **What is the main finding and its importance?**
  Local sweat rates increased linearly with age at torso sites, driven by higher sweat output per gland, while limb sweating did not. Adult‐like patterns emerged at Tanner stage 3, showing a two‐fold torso–limb difference. Despite these physiological shifts, thermal perceptions were unchanged. These findings advance understanding of female thermoregulation and inform age‐appropriate public health and sportswear design.

## 1 INTRODUCTION

Body temperature regulation in humans is an important homeostatic process resulting from a combination of feedback and feedforward mechanisms aimed at maintaining optimal cellular function during thermal challenges, such as heat stress (Mitchell et al., 2025). In adults, following behavioural thermoregulation (Attia, 1984), the production and evaporation of sweat from the skin surface represents the principal and most powerful method of heat loss during exercise and heat stress (Havenith, 1999; Havenith et al., 2008). It is generally believed that the complete maturation of sweating mechanisms in humans may occur throughout puberty. This consideration is largely derived from empirical observations that children present lower whole‐body sweat rates than adults during exercise and heat stress (Bar‐Or, 1998; Drinkwater et al., 1977; Inoue et al., 2004; Meyer et al., 1992). Furthermore, recent sweat mapping studies in children have indicated that regional sweating patterns observed in pre‐pubertal girls (∼8 years old (yo)) do not resemble those observed in women (i.e., >18 yo) (Arlegui et al., 2021; Smith & Havenith, 2012), whereby girls present their highest local sweat rate (LSR) at the extremities (forehead, hands, feet) instead of the torso (the latter being a body region presenting the highest LSR in women) (Blount et al., 2024; Smith & Havenith, 2012).

This perception of a paediatric ‘thermoregulatory disadvantage’, combined with evidence that children may find it challenging to verbalise their thermal perception and comfort (Fabbri, 2024), has shaped public health messaging that emphasises heightened heat vulnerability in children (UKHSA, 2025). In contrast, the American Academy of Paediatrics have argued that youth have an equally effective thermoregulatory ability as healthy adults (Bergeron et al., 2011), challenging the notion of an inherent disadvantage. These divergent views highlight a critical knowledge gap: it remains unclear how and when complete maturation of local sweating mechanisms across the body occurs throughout puberty, and how such maturation relates to age‑dependent differences in sensitivity to thermal discomfort, during both endogenous (i.e., exercise‐mediated) and exogenous (i.e., environment‐mediated) heat stress. This represents a significant fundamental and applied knowledge gap, which hinders the ability to (a) establish critical age thresholds beyond which heat vulnerability may align with that established in adults; and (b) inform the design of interventions, such as age‐appropriate sportswear, which accommodate the sweating and comfort needs of children throughout puberty.

The implications of this knowledge gap are particularly important for the female population, who continue to be largely underrepresented in thermoregulatory studies (Filingeri, Blount, & Valenza, 2024; Filingeri, Blount, & Ward, 2024; Hutchins et al., 2021). This is despite undergoing unique morphological and physiological change – consider menarche and thelarche – which coincide with rising oestrogen levels known to modulate autonomic thermoeffector responses, such as cutaneous vasodilation and sweat secretion, and could therefore contribute to the maturation and regional redistribution of sweating patterns in females (Biro et al., 2008; Breehl & Caban, 2024).

The aim of this study was therefore to investigate how and when complete maturation of local sweating mechanisms across the whole body occurs throughout puberty in females. A secondary aim was to explore how these age‐dependent changes may relate to variations in children's sensitivity to thermal discomfort, during exercise in the heat. Leveraging validated methodologies for exercise prescription that accommodate an unbiased comparison of sweating responses in paediatric and adult populations (Smallcombe et al., 2025), we hypothesised that LSR across the torso may increase to a greater extent than peripheral sites over the limbs, secondary to changes in sweat output per gland, as recently observed at rest (Amano et al., 2025). We also hypothesised that meaningful changes in regional sweating patterns may occur at specific developmental stages associated with hormonal development in females.

## 2 METHODS

### 2.1 Ethical approval

This study was approved by the University of Southampton Ethics Committee (approval no. 99072) and all procedures conformed to the ethical standards set by the *Declaration of Helsinki*. All participants, and guardians of those aged under 18, completed a health screen questionnaire and provided written informed consent, or informed assent for those aged under 18, prior to testing.

### 2.2 Experimental design

This observational cohort study was conducted in a climatic chamber at the University of Southampton between November 2024 and February 2025. Recruitment involved the purposeful sampling of girls from a range of adolescent age groups and comparison with a control group of adult women. Participants underwent one preliminary trial and one experimental trial, separated by >48 h.

### 2.3 Participants

Twenty‐eight healthy 8‐ to 25‐year‐old (yo) females volunteered to participate in this study. Participants were purposefully recruited by age group to accommodate appropriate representation across pubertal development: 8–9 yo (*n *= 4), 10–11 yo (*n *= 4), 12–13 yo (*n *= 6), 14–15 yo (*n *= 6), 16–17 yo (*n *= 4), and 18–25 yo (*n *= 4). Pubertal development was assessed using self‑reported Tanner staging, completed jointly by participants and their parents with the aid of standardised visual and written descriptors. This approach improves accuracy in younger participants and provides a more nuanced indication of physical and physiological maturation than chronological age alone (Breehl & Caban, 2024). Stage 1 represents pre‐puberty and stage 5 indicates adult‐level development. Participant physical and physiological characteristics spanned the full range of Tanner staging, as presented in Table 1. Participants were not heat‐acclimatised and seasonal variation was limited (Amano et al., 2025). They included physically active females (i.e., performing moderate intensity exercise >3 days per week), who were free of any injury or illness. Of those who had begun menarche (*n* = 15), six participants presented irregular periods at the time of the study and nine were distributed across a typical 28‐day menstrual cycle (mean testing day of cycle: 13 ± 4).

**TABLE 1:** Anthropometric, heat exchange and exercise intensity during exercise (*n* = 28).

	All (*n* = 28)	8–9 yo (*n* = 4)	10–11 yo (*n* = 4)	12–13 yo (*n* = 6)	14–15 yo (*n* = 6)	16–17 yo (*n* = 4)	18–25 yo (*n* = 4)
Age (years)	14.1 ± 4.4	8.8 ± 0.5	10.3 ± 0.5	12.7 ± 0.5	14.3 ± 0.5	16.8 ± 0.5	22.5 ± 2.4
Tanner Stage (min–max)	1–5	1	1–3	2–4	3–4	4–5	5
Height (m)	1.60 ± 0.12	1.40 ± 0.09	1.49 ± 0.06	1.60 ± 0.07	1.71 ± 0.03	1.66 ± 0.05	1.68 ± 0.02
Body mass (kg)	54.1 ± 16.8	33.2 ± 2.6	36.9 ± 6.2	54.0 ± 17.8	62.4 ± 10.3	63.5 ± 12.2	70.5 ± 8.3
BSA (m^2^)	1.54 ± 0.28	1.15 ± 0.06	1.25 ± 0.12	1.54 ± 0.23	1.72 ± 0.14	1.70 ± 0.17	1.80 ± 0.10
BSA:Mass (m^2^/kg)	0.030 ± 0.004	0.035 ± 0.002	0.034 ± 0.003	0.030 ± 0.005	0.028 ± 0.002	0.027 ± 0.003	0.026 ± 0.002
Body fat (%)	17.5 ± 4.6	14.4 ± 4.1	14.1 ± 2.9	17.8 ± 6.0	17.5 ± 4.0	20.0 ± 3.9	21.2 ± 2.8
*E*_req_ (W/m^2^)	154 ± 10	157 ± 13	150 ± 9	153 ± 14	157 ± 9	149 ± 9	162 ± 9
*H*_prod_ (W/m^2^)	160 ± 10	164 ± 9	157 ± 10	159 ± 13	162 ± 10	153 ± 5	168 ± 9
*H*_prod_ (W/kg)	4.78 ± 0.75	5.72 ± 0.70	5.40 ± 0.78	4.72 ± 0.64	4.53 ± 0.43	4.16 ± 0.31^*^	4.31 ± 0.49^*^
*H*_prod_ (W)	247 ± 2	189 ± 21	196 ± 6	247 ± 50	280 ± 31^*^ *^#^*	261 ± 35	301 ± 15^*^ *^#^*
Mean *T*_sk_ (°C)	36.3 ± 0.3	36.6 ± 0.2	36.4 ± 0.2	36.1 ± 0.5	36.3 ± 0.2	36.2 ± 0.2	36.2 ± 0.3
∆*T*_Tympanic_ (°C)	0.57 ± 0.27	0.62 ± 0.10	0.50 ± 0.27	0.63 ± 0.25	0.48 ± 0.32	0.68 ± 0.45	0.50 ± 0.16
WBSR (L)	0.21 ± 0.07	0.17 ± 0.09	0.20 ± 0.06	0.17 ± 0.05	0.21 ± 0.07	0.25 ± 0.08	0.28 ± 0.06

*Note*: Values are presented as means ± SD, except for Tanner stage. ^*^Significant difference from 8–9 year olds. ^#^significant difference from 10–11 year olds (*P *< 0.05). Abbreviations: BSA, body surface area; ∆*T*_Tympanic_, delta change in tympanic temperature from start to end of exercise; *E*_req_, rate of evaporation required for heat balance; *H*_prod_, rate of metabolic heat production; *T*_sk_, mean skin temperature during exercise; WBSL, whole body sweat loss; yo, years old.

### 2.4 Preliminary session

Body mass (KERN 150K2DL, Balingen, Germany; accurate to 0.005 kg) and height (Seca 213 Stadiometer, Birmingham, UK) were measured. Body surface area (BSA) was calculated using the Du Bois and Du Bois (1916) equation which is appropriate for use in children and adults (Haycock et al., 1978). Body fat percentage was estimated from skinfold thickness measured at four sites (abdomen, suprailiac, triceps, thigh) (Jackson & Pollock, 1985).

**FIGURE 1:**
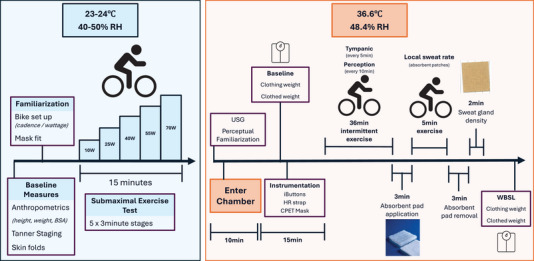
Schematic illustration of experimental design. Preliminary session (left) and experimental session (right).

Participants performed a submaximal incremental exercise test of five 3‐min stages in temperate conditions (air temperature: 24.0 ± 0.2°C; relative humidity: 50.7 ± 7.0%) on a cycle ergometer (Lode Corvial Recumbent, Groningen, Netherlands) (Figure 1). Expired gases were measured via indirect calorimetry (Quark CPET, Cosmed, Rome, Italy) to determine the individual exercise workload required to elicit the target *E*_req_ for the experimental trials, as previously described (Cramer & Jay, 2019). Equations used to calculate partitional calorimetry parameters, including *E*_req_, rate of convective and radiative heat exchange (*C* + *R*), rate of respiratory convective and evaporative heat loss (*C*_res_ + *E*_res_) and *H*_prod_, are presented in Appendix A (Parsons, 2014).

### 2.5 Experimental session

Participants were asked to refrain from strenuous exercise 24 h prior to the experimental trial. Upon arrival they provided a urine sample to measure urine specific gravity (USG) (digital refractometer, KERN, Balingen, Germany) with euhydration confirmed by a USG < 1.025 (Casa et al., 2005). A standardised ensemble of shorts, sports bra and T‐shirt was provided to the participants, and shoes and socks were worn (∼0.3 clo). All clothing items were weighed to the nearest 0.1 mg (PCB 350‐3, KERN).

Prior to entering the climate chamber, participants were familiarised with the whole‐body perceptual assessment scales for thermal sensation (TS), wetness perception (WP) and thermal comfort (TC), leveraging adapted Likert scales for paediatric use (Teli et al., 2013). Likert scales ranged from 1 to 4 (TS: 1 = neutral/OK, 4 = hot; WP: 1 = dry, 4 = dripping wet; TC: 1 = comfortable, 4 = very uncomfortable). Upon entry to the climate chamber (air temperature: 36.6 ± 0.2°C; relative humidity: 48.4 ± 2.2%), clothed body mass was measured (KERN 150K2DL; accurate to 0.005 kg) (Figure 1). Whole‐body sweat loss (WBSL) was calculated by subtracting post‐trial clothed body mass from pre‐trial clothed body mass, corrected for sweat trapped in clothing. This method was used instead of pre and post nude weights for ethical reasons. Participants were then equipped with an optical heart rate (HR) monitor (Verity Sense, POLAR, Kempele, Finland) and seven skin temperature (*T*_sk_) sensors (iButtons, Maxim, San Jose, USA; 0.2 Hz) were fixed on the abdomen, shoulder, upper breast, upper back, lower back, thigh and shin. Weighted mean *T*_sk_ was calculated as the weighted averaged of measurements at the chest (0.3), shoulder (0.3), thigh (0.2) and calf (0.2) (Ramanathan, 1964).

Participants sat for 5 min such that baseline measures of HR, tympanic temperature (*T*_tym_), and perceptual measurements were taken, then they cycled for 36 + 5 min, alternating between 3‐min intervals of higher (*E*_req_: 170 W/m^2^) and lower (*E*_req_: 140 W/m^2^) intensities. Exercise prescribed at a fixed *E*_req_ in W/m^2^ allows for a more unbiased investigation of LSR differences by accounting for biophysical differences in body size, thereby substantially reducing confounding effects (Cramer & Jay, 2015). However, this approach does not fully eliminate maturation‐related differences in metabolic heat production or sudomotor drive which may persist due to differences in lean body mass, muscle composition, and neural factors (Baker, 2017; Hu et al., 2018). ${\dot{V}}_{O_{2}}$, HR and *T*_sk_ were measured continuously throughout exercise. TS, WP and TC were measured at baseline then at 10‐min intervals and *T*_tym_ at 5‐min intervals (Figure 1). *T*_tym_ (ThermoScan 7+, Braun, Bussigny, Switzerland) was used due to ethical restrictions on the use of more invasive techniques, such as rectal sensors or ingestible telemetric pills with children.

Following the initial 36‐min of cycling, participants briefly stopped to allow the set‐up of LSR collection for the final 5‐min of exercise, via the absorbent patch technique (Smith & Havenith, 2012). Prior to the trial, absorbent patches (max absorption = 4655 ± 220 g m^−2^) were cut to size (9 cm^2^), individually sealed in ziplock bags, marked, and weighed to the nearest 0.1 mg (PCB 350‐3, KERN). The skin was dried, then patches were applied using a waterproof film dressing (Tegaderm, 3M, St Paul, MN, USA) to the palmar surface of the hand, bra triangle, abdomen, upper back, lower back, thigh and shin on the contralateral side to the *T*_sk_ sensors. Following application, participants cycled for 5 min, then the patches were removed, sealed in ziplock bags, and weighed. LSR (g m^−2^ h^−1^) was calculated from the relative change in pre‐ to post‐patch weight, divided by the surface area and application time.

Following cessation of exercise and immediately after removal of the LSR pads, heat activated sweat gland density (HASGD) was assessed non‐invasively at the bra triangle site using the modified iodine technique (Gagnon et al., 2012). HASGD was quantified to estimate maturation‐dependent changes in sweat output per gland. The bra triangle site was selected as previous work demonstrated this site to have one of the highest levels of LSR and HASGD across the female torso (Blount et al., 2024). Cotton paper patches were cut to 3 × 3 cm squares and impregnated with iodine. The skin was blotted dry to move excess water, then the iodine paper was firmly pressed to the skin for ∼5 s. Sweat from active sweat glands appeared as small blue dots on the iodine infused paper. The paper was sealed in air‐tight bags and scanned immediately after testing at a high resolution (600 dots/inch) then analysed using ImageJ (http://rsbweb.nih.gov/ij/index.html). This process is explained in detail by Gagnon et al. (2012). Once the images were processed, a sub‐set of images were assessed by two raters to ensure rigor in the analysis (Peel et al., 2022). When raters agreed (<10% variation), the primary investigator's measurement was taken; where disagreement between raters was >10%, an average from the two raters was used. An estimation of sweat output per gland could then be calculated by dividing the sweat rate by the density of heat‐activated sweat glands for the bra triangle site.

### 2.6 Statistical analysis

Statistical analyses were carried out using SPSS (version 28.1; IBM Corp., Armonk, NY, USA). Normality testing using the Shapiro–Wilk test was performed for all datasets, and significance was set at *P *< 0.05. Subsequently, descriptive data are reported as means and standard deviation (SD).

Partitional calorimetry was performed to ensure exercise prescription at a fixed *E*_req_ in W/m^2^ and to assess the relative contribution of evaporative and dry heat exchange in relation to age. One‐way analyses of variance (ANOVA) were performed to assess the impact of age groups on *E*_req_, *H*_prod_ and *T*_sk_ throughout exercise.

To address the primary aim, Pearson correlation analyses were performed to assess the relationships between age and local sweating responses at each skin site (i.e., LSR, heat activated sweat gland density and sweat output per gland)_._ To assess the evolution of regional sweating patterns with ageing/development, skin sites were grouped into torso and extremity sites to allow comparison between central vs. peripheral body regions. Two levels of grouped body sites were selected for two reasons: (i) to investigate the hypothesis that LSR across the torso would increase to a greater extent than the periphery to align with sweating patterns seen in adulthood; and (ii) to consider applications to sportswear, for which one predominantly designs separate clothing items for the torso and lower body. Two separate two‐way ANOVAs were used with the factor of either age groups (6 levels: 8–9, 10–11, 12–13, 14–15, 16–17, 18–25) or Tanner staging (1–5), and body site (2 levels: torso and extremities). When a significant interaction was observed, a *post hoc* analysis for multiple comparisons was conducted using Bonferroni's multiple comparisons test. Categorical analyses by age group/Tanner stage were pre‑specified as secondary, exploratory comparisons given small subgroup sizes and are interpreted cautiously.

The Kruksal–Wallis test was used to investigate the impact of age (6 levels: 8–9, 10–11, 12–13, 14–15, 16–17, 18–25) on perceptual outcomes (TS, WP, TC) at each time point. The effect of time on perceptual outcomes (TS, WP, TC) was analysed using Friedman's test.

## 3 RESULTS

### 3.1 Descriptive exercise, physiological and calorimetric data

Anthropometric, heat exchange and exercise intensity data for all participants are presented in Table 1. Pubertal stage defined by Tanner staging, had a statistically significant positive correlation with age (*r* = 0.85, *P *< 0.001).

As per the study design, there was no statistically significant effect of age group on *E*_req_ (*P *= 0.488) or *H*_prod_ (*P *= 0.383) in W/m^2^, nor on mean *T*_sk_ (*P* = 0.307). By contrast, there was a statistically significant effect of age on: (i) absolute *H*_prod_ in W (*P *< 0.001), with the youngest girls having lower *H*_prod_ in W than adults, and (ii) *H*_prod_ in W/kg (*P *= 0.005), with the youngest girls having greater *H*_prod_ in W/kg than adults (Table 1).

### 3.2 Relationship between age, local sweat rates, activated sweat gland density and sweat gland output

**FIGURE 2:**
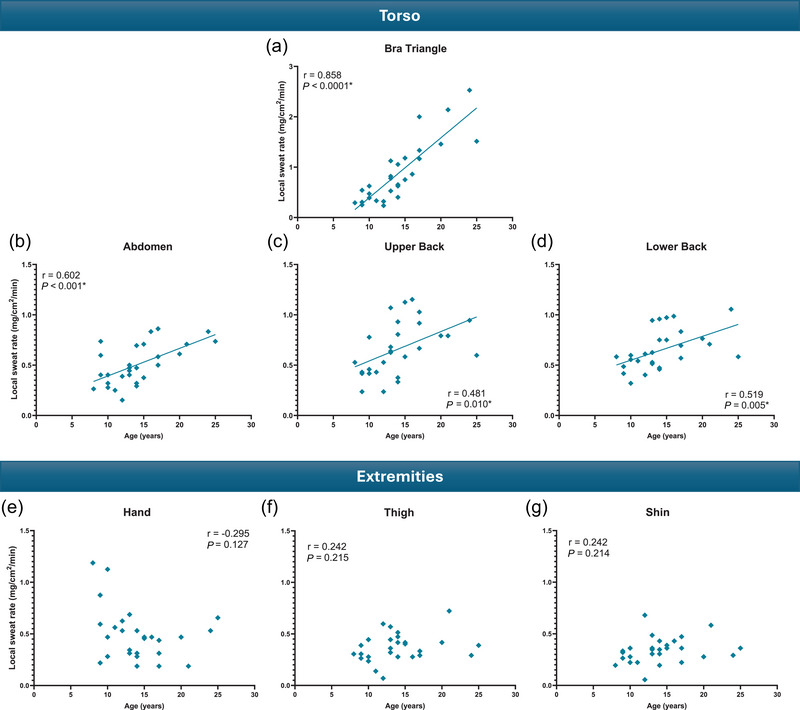
Relationship between age and local sweat rate at the torso sites (bra triangle (a), abdomen (b), upper back (c), lower back (d)) and the extremities (hand (e), thigh (f), shin (g)) (*n* = 28).

A statistically significant positive linear correlation was found between age and LSR at the four torso sites, namely the bra triangle (*r* = 0.858, *P* < 0.0001), abdomen (*r* = 0.602, *P* < 0.001), upper back (*r* = 0.481, *P* = 0.010) and lower back (*r* = 0.519, *P* = 0.005) (Figure 2). In contrast, no statistically significant correlation was found between age and LSR at the extremities, namely the hand (*r* = −0.295, *P* = 0.127), thigh (*r* = 0.242, *P* = 0.215) and shin (*r* = 0.242, *P* = 0.214) (Figure 2).

**FIGURE 3:**
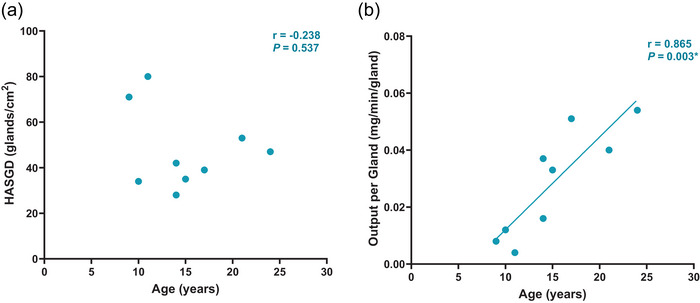
Relationship between age and (a) heat activated sweat gland density (HASGD), and (b) sweat output per gland at the bra triangle (*n* = 9).

Subset analyses of HASGD at the bra triangle (*n* = 9) demonstrated no statistically significant correlations with age (*r* = −0.087, *P* = 0.824). Conversely, estimated sweat output per gland at the bra triangle had a statistically significant positive linear correlation with age (*r* = 0.865, *P* = 0.003) (Figure 3).

### 3.3 Regional patterns of local sweat rates

#### 3.3.1 Age groups

**FIGURE 4:**
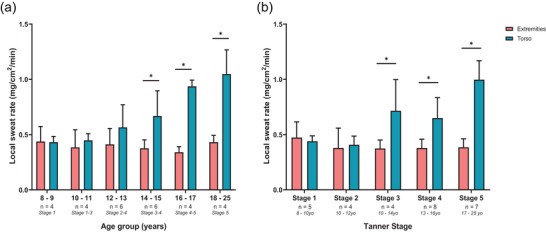
The effect of age (a) and tanner stage (b) on grouped local sweat rates (extremities vs. torso). Presented as means and SD. *Significant difference between sites (*P* ≤ 0.05).

Statistically significant main and interactive effects of age groups and grouped body sites (torso vs. extremities) were observed for LSR (all *P* ≤ 0.001). *Post hoc* analyses of interaction effects indicated no statistically significant differences in LSR between the torso and extremity sites for the 8–9 yo (*P* = 0.956), 10–11 yo (*P* = 0.565) and 12–13 yo (*P* = 0.095) groups. In contrast, a statistically significantly difference in LSR was found at the torso relative to the extremities, such that the torso had a 1.8‐fold higher LSR than the extremities in the 14–15 yo (*P *= 0.003), a 2.8‐fold higher LSR than the extremities in the 16–17 yo (*P *< 0.001), and a 2.4‐fold higher LSR than the extremities in the 18–25 yo (*P *< 0.001) groups (Figure 4a).

#### 3.3.2 Tanner stages

Statistically significant main and interactive effects of Tanner stage and grouped body sites (torso vs. extremities) were observed for LSR (all *P* ≤ 0.001). *Post hoc* analyses of interaction effects indicated no statistically significant differences in LSR between the torso and extremity sites at Tanner stage 1 (*P* = 0.717) and 2 (*P* = 0.786). In contrast, a statistically significantly difference in LSR was found at the torso relative to the extremities, such that the torso had a 1.9‐fold higher LSR than the extremities at stage 3 (*P *= 0.003), a 1.7‐fold higher LSR than the extremities at stage 4 (*P *= 0.001), and a 2.6‐fold higher LSR than the extremities at stage 5 (*P *< 0.001) (Figure 4b).

### 3.4 Perceptual measures

**FIGURE 5:**
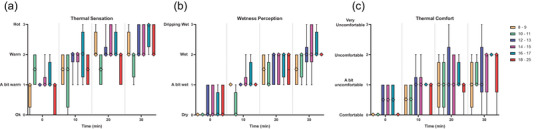
Box plots displaying thermal sensation (a), wetness perception (b) and thermal comfort (c) measured at 4 time points and grouped by age. Data presented as median (diamond symbol), interquartile range (box) and min/max (error bars).

Time was identified as having a statistically significant effect on TS (*P *< 0.001), WP (*P *< 0.001) and TC (*P *< 0.001) (Figure 5). *Post hoc* tests indicated no statistically significant effect of age group at any time point for TS or TC (*P* > 0.05). On average, participants felt ‘a bit warm’ at baseline (median ± interquartile range; 1 ± 0) and increased to between ‘warm’ and ‘hot’ by the end of exercise (2 ± 1). All participants also felt ‘comfortable’ at baseline (0 ± 1) and increased to between ‘a bit uncomfortable’ and ‘uncomfortable’ by the end of exercise (2 ± 1). *Post hoc* tests for WP showed no statistically significant effect of age group at minute 0, 20 and 30 (*P* > 0.05), with all participants feeling ‘dry’ at baseline (0 ± 0.25) and increasing to ‘wet’ by the end of exercise (2 ± 0). However, at minute 10, WP was statistically significantly lower in 10–11 yo (0 ± 0.25) than all other age groups (1 ± 0) (*P* = 0.012).

## 4 DISCUSSION

The aim of this study was to investigate how and when complete maturation of local sweating mechanisms across the whole‐body occurs throughout puberty in females, and how these age‐dependent changes may relate to variations in children's sensitivity to thermal discomfort, during exercise in the heat. In line with our primary hypotheses, our results indicated that LSR across the torso (i.e., chest, abdomen, upper and lower back), but not the limbs (hand, thigh, shin), increased linearly with age (see Figure 2), due to age‐dependent increases in sweat output per gland (see Figure 3b). The transition of regional sweating patterns from children‐like (i.e., higher LSR at extremities than torso) to adult‐like (i.e., higher LSR at torso than extremities) became apparent and meaningful (i.e., 2‐fold difference between torso and limbs) at a specific developmental stage associated with Tanner stage 3 and a chronological age of 14–15 yo (see Figure 4). Our results also indicated that perceptions of temperature, wetness and thermal comfort during exercise in the heat did not differ across age‐groups. Altogether, these findings are novel and important, as they provide the first detailed accounting of the maturation of regional sweating patterns across the body in females exercising in the heat.

### 4.1 Maturation of sweating

The regional sweating patterns observed in our oldest female cohort (i.e., 18–25 yo) aligns with the findings of Smith and Havenith (2012), who reported women (i.e., 21 ± 1 yo) to present higher LSR at the torso than the limbs. Similarly, the regional sweating patterns observed in our youngest female cohort (i.e., 8–9 yo) support the findings of Arlegui et al. (2021), who reported girls (i.e., 8 yo) to present higher LSR at the extremities (forehead, hands, feet) than the rest of the body. Our detailed evaluation of regional sweating patterns across the full pubertal development period in women (i.e., age range 8–25 yo and Tanner stages 1–5) critically extends those previous insights to demonstrate a specific developmental stage associated with sweating maturation in women, who are a group that undergo unique hormonal changes during puberty and throughout the lifespan.

A plausible explanatory framework for the transition in regional sweating patterns around mid‐puberty is endocrine maturation. While we did not measure circulating sex hormones, and thus cannot suggest a mechanistic inference, we did measure Tanner stages, which can act as a developmental marker. Previous evidence has indicated that key hormonal changes occur when girls reach Tanner stage 3, such as a 20‐ to 40‐fold increase in luteinising hormone relative to pre‐pubertal stages, which in turn causes a rise in circulating oestradiol levels for the first time in female development (DiVall & Radovick, 2009; Rosenfield et al., 2013). Increased circulating oestrogens in humans have been demonstrated to increase heat dissipation responses, including cutaneous vasodilation and sweating (Stephenson & Kolka, 1999). Elevated oestrogen levels during the female menstrual cycle, unopposed by progesterone, have also been associated with small decreases in female body temperature, an observation which further highlights the role of oestrogens in modulating heat exchange and body temperature in females (De Mouzon et al., 1984; Marshall, 1963). Our observations did highlight an apparent shift in regional sweating patterns coinciding with Tanner stage 3 in our female cohort, yet it is necessary to add the caveat that the subgroup sizes for this categorical analysis were small (*n* = 4–6) and Tanner staging was self‐reported. However, this generates an interesting hypothesis for future mechanistic studies to investigate to more directly establish causality between the levels of circulating sex hormones during puberty and sweating maturation.

The upregulation of sweat output per gland at the bra triangle during pubertal development in girls observed in this study (see Figure 3b) aligns with recent findings that forearm sweat output per gland also increases in children under cholinergic stimulation at rest (Amano et al., 2025). Because pharmacological cholinergic stimulation bypasses the centrally mediated thermal drive that operates during exercise – and can therefore elicit higher sweat rates (Saltin & Gagge, 1971; Shibasaki & Crandall, 2010) – both approaches suggest that sweat gland output capacity increases with maturation across body sites, though its expression during exercise may be region‐specific. In our cohort, chest subset analyses showed no age‐related change in HASGD, but sweat output per gland increased with age, indicating functional upregulation at this torso site. At limb sites, however, adolescent skin expansion may reduce sweat gland density, consistent with significantly lower forearm gland density with age (Amano et al., 2025). This variation in the extent of skin stretch across different body sites potentially offsets the age‑related gains in sweat output per gland and yields the flat age–LSR relationship we observed at the extremity sites. However, we acknowledge that the subgroup analysis of chest HASGD had a smaller sample size (*n* = 9). Additionally, the absent age–LSR relationship at the hand may reflect distinct sweat control patterns at the hands/feet, as originally proposed by Kuno (1956), and supported by both our findings and prior adult and paediatric sweat mapping studies (Arlegui et al., 2021; Smith & Havenith, 2012). Although neither this study, nor Amano et al. (2025) was designed to interrogate the mechanisms underlying the regulation of sweat output per gland, similar hypotheses can be proposed: age‐related changes may arise from anatomical maturation of the sweat glands (e.g., duct length, secretory coil area) (Sato & Sato, 1983), and gross body morphological development causes variation in sweat gland density, and potentially an increased exposure to circulating growth and sex hormones, for which eccrine sweat glands express receptors (Choudhry et al., 1992; Lobie et al., 1990; Pelletier & Ren, 2004).

From a methodological standpoint, the present study provides a more rigorous mechanistic evaluation of age‐dependent change in LSR patterns during exercise than previous research to date. For example, Smith and Havenith (2012) and Arlegui et al. (2021) leveraged experimental models using exercise prescribed at a fixed percentage of maximal HR or ${\dot{V}}_{O_{2}\max}$ to study LSR patterns. Conversely, our study adopted an exercise prescription designed to achieve equal levels of *E*_req_ in W/m^2^ across all age groups (see Table 1). This is because individual variations in *E*_req_ in W/m^2^ (which can occur when exercise is prescribed at a fixed percentage of maximal HR or ${\dot{V}}_{O_{2}\max}$) have been demonstrated to explain a large proportion of variance in LSR when comparing groups that differ in size and surface area (Cramer & Jay, 2014; Cramer & Jay, 2015). Nonetheless, fixed *E*_req_ prescription cannot guarantee independence from maturation‐related differences in metabolic heat production or neural drive to sweat which can also be influenced by lean mass, and muscle composition may persist (Baker, 2017; Hu et al., 2018).

### 4.2 Maturation of thermal perception

Our observations on the maturation of sweating in girls, and their relevance for our basic understanding of autonomic body temperature regulation, is well complemented by our results on the perceptual responses of our female cohort during exercise in the heat. This is particularly important given the paucity of evidence on thermal sensation of children in the heat. Inoue et al. (2009) has previously observed similar thermal sensations between boys (9–11 yo) and men during passive heat exposure (from 28 to 40°C), for the same change in *T*_sk_. Our results extend these findings to exercising females, as our girls presented equal thermal sensations and discomfort to adult females under exercising conditions resulting in similar elevations in mean *T*_sk_ (i.e., ∼36°C – see Table 1). These observations indicate that younger girls are likely to be equally sensitive to warmth and discomfort as their older counterparts. Furthermore, our findings indicated that perceptions of whole‐body wetness during exercise in the heat did not differ across age groups. One could speculate that this provides evidence that younger girls may have a greater wetness sensitivity than older girls as this same magnitude of perceived wetness in the younger girls occurred in the presence of much lower LSR at certain body sites. This theory may not be entirely speculative, as our group has recently observed boys and girls aged 7–12 years old to report greater wetness perceptions than adults during the application of the same local wet stimuli onto their skin while at rest (Valenza et al., 2026). However, future studies would have to investigate further whether this potential age‐dependent variation in whole body wetness perception in girls is indeed robust and whether this impacts meaningful age‐dependent differences in thermal behaviours under a paradigm that allows free behaviour (e.g., cool‐seeking, drinking, etc.) (Schlader & Vargas, 2019).

### 4.3 Public health and industrial applications

Beside their relevance to understand the basic biological mechanisms underlying sweating maturation and perception in females, we believe that our findings have important implications for public health and industrial applications.

From a public health messaging standpoint, the American Academy of Paediatrics (Bergeron et al., 2011) has recently issued a position statement suggesting youth have an equally effective thermoregulatory ability as healthy adults and therefore are not at a ‘thermoregulatory disadvantage’ during exercise in the heat as previous research suggested (Davies, 1981; Drinkwater et al., 1977; Meyer et al., 1992; Rivera‐Brown et al., 2006; Wilk et al., 2013). Our observations on the presence of lower LSRs across the torso in our youngest girls, secondary to lower sweat output per gland and despite the same *E*_req_ in W/m^2^, may at first appear in contrast with the American Academy of Paediatrics’ position, i.e., those younger girls were locally secreting less sweat than needed to meet the evaporative requirements for heat balance. Yet, despite these lower LSRs across the torso, and the fact that these younger girls were exercising at a greater level of heat production per unit of body mass than the older girls (i.e., 5.72 vs. 4.31 W/kg – see Table 1), they did not develop a greater change in core (tympanic) temperature than their older counterparts (see Table 1). This observation aligns with recent evidence from Smallcombe et al. (2025), who also found children aged 10–16 to be at no greater risk of hyperthermia than adults during exercise in ambient temperature of up to 40°C. We speculate that this apparently effective maintenance of heat balance in children, despite developing sweating mechanisms, may be supported by a body morphology (e.g., higher body surface area to mass ratio – see Table 1), which could favour heat distribution across the body and dissipation with the environment.

From an industrial standpoint, our observation of a critical age for changes in regional sweating patterns in girls has important applied implications for the sportswear industry, which leverages knowledge of LSR patterns to guide the design of clothing that satisfies the thermal requirements of the user. It is important to note that clothing is commonly designed based on age ranges for specific consumer groups (i.e., kids vs. adults), rather than stages of hormonal development (e.g., Tanner stages). As age and tanner stages were strongly correlated in our study cohort, it allowed us to consider the critical stage for changes in regional sweating patterns in girls as a function of chronological age (see Figure 3a). This analysis indicated that an ∼2‐fold difference in LSR between torso and extremities became apparent in the age group 14–15 yo, which included girls spanning Tanner stages 3 to 4. Consequently, we believe that the meaningful shift in regional sweating patterns observed at 14 yo could inform design considerations such as the need for greater moisture management for the torso vs. limbs from this age onwards, to help maximise comfort for adolescent girls. This approach may have broader benefits, such as improving exercise participation at a time when factors such as body appearance (e.g., perceived sweatiness) may in part contribute to sport drop‐out in girls (Eime et al., 2016; Ogden et al., 2025). This may ultimately increase clothing comfort and performance, thus reducing barriers to participation in sport for women of all ages in a warming climate.

### 4.4 Limitations

There are limitations to this study. First, our study cannot establish the extent by which the observed differences in LSRs in younger vs. older girls may differentially impact the maintenance of heat balance at both lower ambient temperatures than we tested and under greater thermal stress, i.e., uncompensable heat stress. Indeed, previous research has indicated that children may have an increased dependence on dry heat loss mechanisms to compensate for a less developed sweating capacity (Davies, 1981; Drinkwater et al., 1977). Our experiment was designed to maximise the reliance on evaporative heat exchange and minimise reliance on dry heat exchange, which was achieved by setting the environmental temperature to a level equal to skin temperature during exercise (i.e., 36°C). Future studies may therefore consider exposure to ambient conditions that may highlight a ‘thermoregulatory advantage’ of children under less warm conditions, where the larger body surface area to mass ratio of younger children may favour dry heat exchange mechanisms and facilitate water conservation. Future studies may also consider different exercise modalities such as running, which may facilitate achieving greater levels of heat production than cycling and associated sweating requirements. We indeed noted in our investigation that the absolute level of cycling workload was primarily limited by the level of leg strength of our youngest participants (i.e., higher workloads to induce higher thermal loads were not achievable due to early onset fatigue). In addition, the use of tympanic temperature as an indirect measure of core temperature likely resulted in a conservative estimation of internal heat strain, as this index is known to underestimate core temperature relative to more direct measures (i.e., rectal or gastro‐intestinal pills) (Huggins et al., 2012). Finally, HASGD represents an instantaneous snapshot of active glands, whereas LSR was integrated over 5 min; thus, age‐related differences in pulsatile activation frequency could add variance to the LSR/HASGD estimate. To minimise timing mismatch, HASGD imaging was performed immediately after LSR collection, and the iodine imprint duration was standardised (∼ 5 s) using the modified iodine technique, but future work could consider duplication of HASGD imaging.

### 4.5 Conclusions

Our study provides the first detailed account of the maturation of regional sweating patterns across the female body, a commonly under‐represented cohort in thermoregulatory studies. We provide novel evidence that the maturation of regional patterns of LSR occurs primarily because of linear increases in LSR across the torso but not the limbs, and at a particular stage of maturation, which we identified to coincide with Tanner stage 3 and 14–15 years of age. We also provide evidence that perceptions of temperature, wetness and thermal comfort during exercise in the heat did not differ across age‐groups. Our results advance our fundamental understanding of the maturation of sweating in females, and they also have important applied implications for person‐centred public health messaging and sportswear design.

## AUTHOR CONTRIBUTIONS

Hannah Blount, Davide Filingeri, Grant Simmons and Peter Worsley conceived the initial outline for the article. Acquisition was completed by Hannah Blount, Jade Ward and Nuno Koch Esteves. Hannah Blount, Davide Filingeri, and Peter Worsley contributed to interpretation. Hannah Blount, Davide Filingeri, Grant Simmons and Peter Worsley contributed to drafting the manuscript. All authors approved the final version of the manuscript and agree to be accountable for all aspects of the work in ensuring that questions related to the accuracy or integrity of any part of the work are appropriately investigated and resolved. All persons designated as authors qualify for authorship and only those who qualify for authorship are listed.

## CONFLICT OF INTEREST

The authors declare they have no conflicts of interest.

## Data Availability

Data will be made available upon publication at the University of Southampton data repository (PURE; URL to be activated upon publication).

## APPENDIX A

### CALCULATIONS

#### Calculation 1. Body surface area (Du Bois & Du Bois, 1916)

$$\mathrm{BSA}=Wt\;{\left(\mathrm{kg}\right)}^{0.425}\times \mathrm{Ht}\;{\left(\mathrm{cm}\right)}^{0.725}\times 0.007184.$$

#### Calculation 2. Body fat percentage (Jackson & Pollock, 1985)

$$\begin{array}{ccc} \mathrm{BF}\% & = & \left(0.29669\times \mathrm{sum}\;\mathrm{of}\;\mathrm{skinfolds}\right)-\left[0.00043\times {\left(\mathrm{sum}\mathrm{of}\mathrm{skinfolds}\right)}^{2}\right]\\ & & +\;(0.02963\times \mathrm{age})+1.4072 \end{array}$$

#### Calculation 3. Rate of evaporation required for heat balance (*E*_req_) (Parsons, 2014)

$$E_{\mathrm{req}=}H_{\mathrm{prod}}-\left(C_{sk}+R_{sk}\right)-\left(C_{\mathrm{res}}+R_{res}\right)\;\left[W/m^{2}\right]$$

where the rate of metabolic heat production (*H*_prod_) is calculated as:

$$H_{\mathrm{prod}}=M-Wk\;\left[W/m^{2}\right]$$

where *W* is the rate of mechanical work (i.e., power output; *W*) and *M* is the metabolic rate, which was estimated by measuring the rate of oxygen consumption and carbon dioxide production using the equation:

$$M={\dot{V}}_{O_{2}}\times \;\frac{\left[\left(\frac{\mathrm{RER}-0.7}{0.3}\right)\times e_{c}\right]+\left[\left(\frac{1.0-\mathrm{RER}}{0.3}\right)\times e_{f}\right]\;}{60\;\times \mathrm{BSA}}\times 1000$$

where *e*_c_ is the caloric equivalent per litre of oxygen for the oxidation of carbohydrates (21.13 kJ), and e_f_ is the oxidation of fat (19.62 kJ) (Nishi, 1981).

The rate of dry heat exchange from the skin (*C*_sk_ + *R*_sk_) is calculated as the sum of convective (*C*) and radiative (*R*) heat transfer from the skin using the equation:

$$C_{\mathrm{sk}}+R_{\mathrm{sk}}=\frac{T_{\mathrm{sk}}-T_{o}}{R_{\mathrm{cl}}+\left(\frac{1}{f_{\mathrm{cl}}\cdot h}\right)}$$

*T*_sk_ is skin temperature and *T*_o_ is operative temperature:

$$T_{o}=\frac{h_{r}T_{r}+h_{c}T_{a}}{h_{r}+h_{c}}$$

*R*_cl_ is the dry heat transfer resistance of clothing and was calculated as:

where *I*_cl is_ 0.3 (Tang et al., 2022). *h* is the combined heat transfer coefficient (*h*_c_ + *h*_r_) where *h*_r_ is the radiative heat transfer coefficient estimated using:

$$h_{r}=4\varepsilon \sigma \frac{A_{r}}{A_{D}}{\left[273.2+\frac{T_{\mathrm{sk}}+T_{r}}{2}\right]}^{3}\;\left[W/m^{2}/K\right]$$

ɛ is the emissivity of the body surface (0.95), σ is the Stefan–Boltzmann constant (5.67 × 10^−8^ W m^−2^ K^−1^), *A*_r_/*A*_D_ is the effective radiative area of the body (0.7 m^2^) for a sitting person, and *T*_r_ is the mean radiant temperature (°C) which was equivalent to the dry‐bulb temperature as tests were conducted indoors.

*C*_res_ + *E*_res_ is respiratory heat exchange and was calculated as the sum of convective (*C*) and radiative (*R*) heat transfer from respiration using the equation:

$$\begin{array}{ccc} C_{\mathrm{res}}+E_{\mathrm{res}} & = & (0.001516\times M\times \left(28.56+0.641P_{a}-0.885T_{a}\right)\\ & & +\;\left[0.00127\times M\times \left(59.34+0.53T_{a}-11.63P_{a}\right)\right]\left[W/m^{2}\right] \end{array}$$

where *P*_a_ is the ambient water vapor pressure (kPa).

## References

[eph70286-bib-0001] Amano, T. , Yasuda, S. , Yokoyama, S. , Oshima, S. , Okamoto, Y. , Otsuka, J. , Kato, H. , Kunimasa, Y. , Hiwa, T. , Fujii, N. , Kenny, G. P. , Hosokawa, Y. , Mündel, T. , Kondo, N. , & Inoue, Y. (2025). Biological maturation and sex differences of cholinergic sweating in prepubertal children to young adults. Annals of the New York Academy of Sciences, 1547(1), 183–191. 40233267 10.1111/nyas.15331

[eph70286-bib-0002] Arlegui, L. , Smallcombe, J. W. , Fournet, D. , Tolfrey, K. , & Havenith, G. (2021). Body mapping of sweating patterns of pre‐pubertal children during intermittent exercise in a warm environment. European Journal of Applied Physiology, 121(12), 3561–3576. 34549334 10.1007/s00421-021-04811-4 PMC8571233

[eph70286-bib-0003] Attia, M. (1984). Thermal pleasantness and temperature regulation in man. Neuroscience and Biobehavioral Reviews, 8(3), 335–342. 6504416 10.1016/0149-7634(84)90056-3

[eph70286-bib-0004] Baker, L. B. (2017). Sweating rate and sweat sodium concentration in athletes: A review of methodology and intra/interindividual variability. Sports Medicine, 47(S1), 111–128. 28332116 10.1007/s40279-017-0691-5 PMC5371639

[eph70286-bib-0005] Bar‐Or, O. (1998). Effects of age and gender on sweating pattern during exercise. International Journal of Sports Medicine, 19(S 2), S106–S107. 9694411 10.1055/s-2007-971970

[eph70286-bib-0006] Bergeron, M. F. , DiLaura Devore, C. , & Rice, S. G. , Council On Sports Medicine and Fitness and Council on School Health . (2011). Climatic heat stress and exercising children and adolescents. Pediatrics, 128(3), e741–e747. 21824876 10.1542/peds.2011-1664

[eph70286-bib-0007] Biro, F. M. , Huang, B. , Daniels, S. R. , & Lucky, A. W. (2008). Pubarche as well as thelarche may be a marker for the onset of puberty. Journal of Pediatric and Adolescent Gynecology, 21(6), 323–328. 19064225 10.1016/j.jpag.2007.09.008 PMC3576862

[eph70286-bib-0008] Blount, H. , Valenza, A. , Ward, J. , Caggiari, S. , Worsley, P. R. , & Filingeri, D. (2024). The effect of female breast surface area on heat‐activated sweat gland density and output. Experimental Physiology, 109(8), 1330–1340. 38847458 10.1113/EP091850 PMC11291870

[eph70286-bib-0009] Breehl, L. , & Caban, O. (2024). Physiology, Puberty. In StatPearls. StatPearls Publishing. 30521248

[eph70286-bib-0011] Casa, D. J. , Clarkson, P. M. , & Roberts, W. O. (2005). American College of Sports Medicine roundtable on hydration and physical activity: Consensus statements. Current Sports Medicine Reports, 4(3), 115–127. 15907263 10.1097/01.csmr.0000306194.67241.76

[eph70286-bib-0012] Choudhry, R. , Hodgins, M. B. , Van der Kwast, T. H. , Brinkmann, A. O. , & Boersma, W. J. (1992). Localization of androgen receptors in human skin by immunohistochemistry: Implications for the hormonal regulation of hair growth, sebaceous glands and sweat glands. Journal of Endocrinology, 133(3), 467–75. 1613448 10.1677/joe.0.1330467

[eph70286-bib-0013] Cramer, M. N. , & Jay, O. (2014). Selecting the correct exercise intensity for unbiased comparisons of thermoregulatory responses between groups of different mass and surface area. Journal of Applied Physiology, 116(9), 1123–1132. 24505102 10.1152/japplphysiol.01312.2013

[eph70286-bib-0014] Cramer, M. N. , & Jay, O. (2015). Explained variance in the thermoregulatory responses to exercise: The independent roles of biophysical and fitness/fatness‐related factors. Journal of Applied Physiology, 119(9), 982–989. 26316511 10.1152/japplphysiol.00281.2015 PMC4628991

[eph70286-bib-0015] Cramer, M. N. , & Jay, O. (2019). Partitional calorimetry. Journal of Applied Physiology, 126(2), 267–277. 30496710 10.1152/japplphysiol.00191.2018 PMC6397408

[eph70286-bib-0016] Davies, C. T. (1981). Thermal responses to exercise in children. Ergonomics, 24(1), 55–61. 7227361 10.1080/00140138108924830

[eph70286-bib-0017] De Mouzon, J. , Testart, J. , Lefevre, B. , Pouly, J.‐L. , & Frydman, R. (1984). Time relationships between basal body temperature and ovulation or plasma progestins. Fertility and Sterility, 41(2), 254–259. 6421622 10.1016/s0015-0282(16)47600-4

[eph70286-bib-0018] DiVall, S. A. , & Radovick, S. (2009). Endocrinology of female puberty. Current Opinion in Endocrinology, Diabetes and Obesity, 16(1), 1–4. 19115519 10.1097/med.0b013e3283207937

[eph70286-bib-0019] Drinkwater, B. L. , Kupprat, I. C. , Denton, J. E. , Crist, J. L. , & Horvath, S. M. (1977). Response of prepubertal girls and college women to work in the heat. Journal of Applied Physiology‐Respiratory, Environmental and Exercise Physiology, 43(6), 1046–1053. 606689 10.1152/jappl.1977.43.6.1046

[eph70286-bib-0020] Du Bois, D. , & Du Bois, E. F. (1916). Clinical calorimetry: Tenth paper a formula to estimate the approximate surface area if height and weight be known. Archives of Internal Medicine, XVII(6_2), 863–871.

[eph70286-bib-0021] Eime, R. M. , Harvey, J. T. , Charity, M. J. , Casey, M. M. , Westerbeek, H. , & Payne, W. R. (2016). Age profiles of sport participants. BMC Sports Science, Medicine and Rehabilitation, 8(1), 6. 10.1186/s13102-016-0031-3 PMC4788892 26973792

[eph70286-bib-0022] Fabbri, K. (2024). The thermal comfort and child development psychology. In K. Fabbri (Ed.), Thermal comfort perception: A questionnaire approach focusing on children (pp. 157–185). Springer International Publishing. 10.1007/978-3-031-52610-7_6

[eph70286-bib-0023] Filingeri, D. , Blount, H. , & Valenza, A. (2024). Female thermal sensitivity and behaviour across the lifespan: A unique journey. Experimental Physiology, 110(2), 191–195. 38451148 10.1113/EP091454 PMC11782171

[eph70286-bib-0024] Filingeri, D. , Blount, H. , & Ward, J. (2024). Thermal physiology is a (wo)man's world! The Journal of Physiology, 602(5), 769–770. 38421340 10.1113/JP286333

[eph70286-bib-0025] Gagnon, D. , Ganio, M. S. , Lucas, R. A. , Pearson, J. , Crandall, C. G. , & Kenny, G. P. (2012). Modified iodine‐paper technique for the standardized determination of sweat gland activation. Journal of Applied Physiology, 112(8), 1419–1425. 22323650 10.1152/japplphysiol.01508.2011 PMC3331585

[eph70286-bib-0026] Havenith, G. (1999). Heat balance when wearing protective clothing. Annals of Occupational Hygiene, 43(5), 289–296. 10481628

[eph70286-bib-0027] Havenith, G. , Fogarty, A. , Bartlett, R. , Smith, C. J. , & Ventenat, V. (2008). Male and female upper body sweat distribution during running measured with technical absorbents. European Journal of Applied Physiology, 104(2), 245–255. 18064483 10.1007/s00421-007-0636-z

[eph70286-bib-0028] Haycock, G. B. , Schwartz, G. J. , & Wisotsky, D. H. (1978). Geometric method for measuring body surface area: A height‐weight formula validated in infants, children, and adults. Journal of Pediatrics, 93(1), 62–66. 650346 10.1016/s0022-3476(78)80601-5

[eph70286-bib-0029] Hu, Y. , Converse, C. , Lyons, M. , & Hsu, W. (2018). Neural control of sweat secretion: A review. British Journal of Dermatology, 178(6), 1246–1256. 28714085 10.1111/bjd.15808

[eph70286-bib-0030] Huggins, R. , Glaviano, N. , Negishi, N. , Casa, D. J. , & Hertel, J. (2012). Comparison of rectal and aural core body temperature thermometry in hyperthermic, exercising individuals: A meta‐analysis. Journal of Athletic Training, 47(3), 329–338. 22892415 10.4085/1062-6050-47.3.09 PMC3392164

[eph70286-bib-0031] Hutchins, K. P. , Borg, D. N. , Bach, A. J. E. , Bon, J. J. , Minett, G. M. , & Stewart, I. B. (2021). Female (Under) representation in exercise thermoregulation research. Sports Medicine—Open, 7(1), 43. 34156570 10.1186/s40798-021-00334-6 PMC8219822

[eph70286-bib-0032] Inoue, Y. , Ichinose‐Kuwahara, T. , Nakamura, S. , Ueda, H. , Yasumatsu, H. , Kondo, N. , & Araki, T. (2009). Cutaneous vasodilation response to a linear increase in air temperature from 28°C to 40°C in prepubertal boys and young men. Journal of Physiological Anthropology, 28(3), 137–144. 19483375 10.2114/jpa2.28.137

[eph70286-bib-0033] Inoue, Y. , Kuwahara, T. , & Araki, T. (2004). Maturation‐ and aging‐related changes in heat loss effector function. Journal of Physiological Anthropology and Applied Human Science, 23(6), 289–294. 15599077 10.2114/jpa.23.289

[eph70286-bib-0034] Jackson, A. S. , & Pollock, M. L. (1985). Practical Assessment of Body Composition. Physician and Sportsmedicine, 13(5), 76–90. 10.1080/00913847.1985.11708790 27463295

[eph70286-bib-0035] Kuno, Y. (1956). Human perspiration. Thomas, Spring‐filed.

[eph70286-bib-0036] Lobie, P. E. , Breipohl, W. , Lincoln, D. T. , García‐Aragón, J. , & Waters, M. J. (1990). Localization of the growth hormone receptor/binding protein in skin. Journal of Endocrinology, 126(3), 467–71. 2212936 10.1677/joe.0.1260467

[eph70286-bib-0037] Marshall, J. (1963). Thermal changes in the normal menstrual cycle. British Medical Journal, 1(5323), 102–104. 20789592 10.1136/bmj.1.5323.102 PMC2122557

[eph70286-bib-0038] Meyer, F. , Bar‐Or, O. , MacDougall, D. , & Heigenhauser, G. J. (1992). Sweat electrolyte loss during exercise in the heat: Effects of gender and maturation. Medicine & Science in Sports & Exercise, 24(7), 776–781. 1501562

[eph70286-bib-0039] Mitchell, D. , Maloney, S. K. , Snelling, E. P. , Hetem, R. S. , & Fuller, A. (2025). Revisiting concepts of thermal physiology: Understanding feedback and feedforward control, and local temperature regulation. Acta Physiologica, 241(7), e70063. 40452320 10.1111/apha.70063 PMC12127788

[eph70286-bib-0040] Nishi, Y. (1981). Chapter 2 Measurement of Thermal Balance of Man. In K. Cena & J. A. Clark (Eds.), Studies in environmental science (Vol., 10, pp. 29–39). Elsevier.

[eph70286-bib-0041] Ogden, J. , McCourt, A. , & Morgan, R. (2025). ‘You're basically naked’: A qualitative study of why girls drop out of sport in their teenage years. Cogent Psychology, 12(1), 2516316.

[eph70286-bib-0042] Parsons, K. (2014). Human thermal environments: The effects of hot, moderate, and cold environments on human health, comfort, and performance, 3rd ed. CRC Press. 10.1201/b16750

[eph70286-bib-0043] Peel, J. S. , McNarry, M. A. , Heffernan, S. M. , Nevola, V. R. , Kilduff, L. P. , & Waldron, M. (2022). Measurement of thermal sweating at rest and steady‐state exercise in healthy adults: Inter‐day reliability and relationships with components of partitional calorimetry. PLoS ONE, 17(12), e0278652. 36455061 10.1371/journal.pone.0278652 PMC9714830

[eph70286-bib-0044] Pelletier, G. , & Ren, L. (2004). Localization of sex steroid receptors in human skin. Histol Histopathol, 19(2), 629–636. 15024720 10.14670/HH-19.629

[eph70286-bib-0045] Ramanathan, N. (1964). A new weighting system for mean surface temperature of the human body. Journal of Applied Physiology, 19(3), 531–533. 14173555 10.1152/jappl.1964.19.3.531

[eph70286-bib-0046] Rivera‐Brown, A. M. , Rowland, T. W. , Ramirez‐Marrero, F. A. , Santacana, G. , & Vann, A. (2006). Exercise tolerance in a hot and humid climate in heat‐acclimatized girls and women. International Journal of Sports Medicine, 27(12), 943–950. 16739090 10.1055/s-2006-923863

[eph70286-bib-0047] Rosenfield, R. L. , Bordini, B. , & Yu, C. (2013). Comparison of detection of normal puberty in girls by a hormonal sleep test and a gonadotropin‐releasing hormone agonist test. Journal of Clinical Endocrinology & Metabolism, 98(4), 1591–1601. 23457407 10.1210/jc.2012-4136 PMC3615202

[eph70286-bib-0048] Saltin, B. , & Gagge, A. (1971). Sweating and body temperatures during exercise. International Journal of Biometeorology, 15(2–4), 189–194. 5146806 10.1007/BF01803896

[eph70286-bib-0049] Sato, K. , & Sato, F. (1983). Individual variations in structure and function of human eccrine sweat gland. American Journal of Physiology, 245(2), R203–R208. 6881378 10.1152/ajpregu.1983.245.2.R203

[eph70286-bib-0050] Schlader, Z. J. , & Vargas, N. T. (2019). Regulation of body temperature by autonomic and behavioral thermoeffectors. Exercise and Sport Sciences Reviews, 47(2), 116–126. 30632999 10.1249/JES.0000000000000180

[eph70286-bib-0051] Shibasaki, M. , & Crandall, C. G. (2010). Mechanisms and controllers of eccrine sweating in humans. Frontiers in Bioscience‐Scholar, 2(2), 685–696. 10.2741/s94 PMC2866164 20036977

[eph70286-bib-0052] Smallcombe, J. W. , Topham, T. H. , Brown, H. A. , Tiong, M. , Clark, B. , Broderick, C. , Chalmers, S. , Orchard, J. , Mavros, Y. , Périard, J. D. , & Jay, O. (2025). Thermoregulation and dehydration in children and youth exercising in extreme heat compared with adults. British Journal of Sports Medicine, 59(16), 1151–1159. 40514198 10.1136/bjsports-2025-109832 PMC12320596

[eph70286-bib-0053] Smith, C. J. , & Havenith, G. (2012). Body Mapping of sweating patterns in athletes: A sex comparison. Medicine & Science in Sports & Exercise, 44(12), 2350–2361. 22811031 10.1249/MSS.0b013e318267b0c4

[eph70286-bib-0054] Stephenson, L. A. , & Kolka, M. A. (1999). Esophageal temperature threshold for sweating decreases before ovulation in premenopausal women. Journal of Applied Physiology, 86(1), 22–28. 9887109 10.1152/jappl.1999.86.1.22

[eph70286-bib-0055] Tang, Y. , Su, Z. , Yu, H. , Zhang, K. , Li, C. , & Ye, H. (2022). A database of clothing overall and local insulation and prediction models for estimating ensembles’ insulation. Building and Environment, 207(Part A), 108418.

[eph70286-bib-0056] Teli, D. , James, P. A. B. , & Jentsch, M. F. (2013). Thermal comfort in naturally ventilated primary school classrooms. Building Research & Information, 41(3), 301–316.

[eph70286-bib-0057] UKHSA . (2025). Hot weather and heatwaves: Guidance for schools and other education settings. https://educationhub.blog.gov.uk/2025/06/hot‐weather‐and‐heatwaves‐guidance‐for‐schools‐and‐other‐education‐settings/

[eph70286-bib-0058] Valenza, A. , Blount, H. , Ward, J. , Merrick, C. , Wootten, R. , Dearden, J. , Wildgoose, C. , Bianco, A. , Buoite‐Stella, A. , Filingeri, V. L. , Worsley, P. R. , & Filingeri, D. (2026). Skin wetness perception across body sites in children and adolescents aged 7–16 years old. Experimental Physiology, 111(2), 426–434. 40448663 10.1113/EP092691 PMC12857481

[eph70286-bib-0059] Wilk, B. , Pender, N. , Volterman, K. , Bar‐Or, O. , & Timmons, B. W. (2013). Influence of pubertal stage on local sweating patterns of girls exercising in the heat. Pediatric Exercise Science, 25(2), 212–220. 23749395 10.1123/pes.25.2.212

